# Osteoarticular reconstructive surgery in malignant bone tumors: The importance of external fixators

**Published:** 2008-08-15

**Authors:** Burnei Gheorghe, Burnei Cristian, Hodorogea Dan, Gavriliu Stefan, Georgescu Ileana, Vlad Costel

**Affiliations:** * Emergency Clinical “M.S. Curie”, UMF “Carol Davila”, Bucharest; ** Emergency Clinical Hospital, UMF “Carol Davila”, Bucharest

**Keywords:** malignant bone tumors, osteoarticular reconstruction, external fixators

## Abstract

This paper is a retrospective study on 8 patients admitted and treated in Paediatric Surgery and Orthopaedics Clinic of “M. S. Curie” Hospital Bucharest between 1997 and 2007.

The patients with malignant bone tumors (table 1.) were studied by sex, tumor type, location, age at the moment of diagnosis, age at the moment of the last evaluation, type of surgery, external fixator implanted, complications, results and survival period. We also considered for each patient the extent of the tumor to diaphysis, soft tissue involvement, involvement of physis and epiphyseal invasion, articular extent, vessels and nerves invasion, presence of metastases and local skin invasion. The certain diagnosis was based on pathological anatomy exam, because clinical and imagistic data were not decisive in each case.

There were studied only those patients who received external fixators, the only method to achieve oncological safe resection and osteoarticular recontruction. We used monoplanar or circular fixators, in adjustable or mixed mountings.

The postoperative complications were not fatal. The survival period has been between 6 months and 18 years. Only two patients, who have survived 6 months and respectively 18 months, were not able to return to prior activities. The other six were reinserted in social activities.

Nowadays, there is made a great effort to save the affected limbs. The conservative treatment is preferred to the amputation, which is being used in very few cases. The development of reconstructive bone surgery is sustained by the possibility to delineate the tumor by diagnosis based on imaging and by the possibility to use modern preoperative and postoperative chemotherapy and radiotherapy.

Limb conservation was possible only in aggressive benign tumors up to 1970. Since then the same treatment was preferred also in malignant bone tumors, because the relapse appeared as frequent as in cases with amputation but the physical and psychological comfort made the patients to accept it readily.

The goal of malignant bone tumors treatment is to save the life of the patient, to preserve the affected limb, to maintain the length and function of the limb.

Oncologic surgery consists of “en bloc” tumor resection followed by bone reconstruction or modular prosthetic replacement. Modular prosthetic replacement leads to the loss of at least one growing cartilage. The use of radiotherapy in some cases may also affect other growing cartilages, leading to limb length discrepancies.

## Materials and methods

**Case 1**

**Fig.1:**
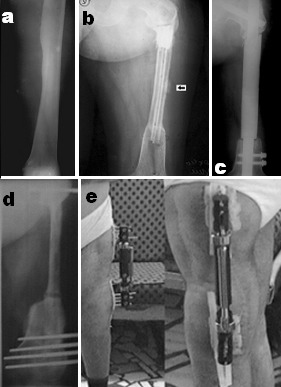
a.Juxtacortical osteosarcoma of the left femur. b. Local relapse. Osteolysis in the mid segment of the fibular grafts and a local bony dense reaction. c. Diaphyseal modular prosthesis on a gamma nail splint. d. Tibial autogenous bone graft on an Ortofix external fixator with delayed union in the distal bone jonction, next healed by compaction. e. 18 years after surgical removal of the tumor, during the bearing of the external fixator, the patient being fully integrated in family and society, even being able to drive his own car.

Patient U.L., male, now aged 34 years, has been diagnosed with left femoral diaphysis juxtacortical (parosteal) osteosarcoma and the diagnosis has been confirmed by pathological anatomy exam (**Fig. 1.a**). Surgical treatment consisted in oncological “en bloc” tumoral resection and bone reconstruction using two autogenous fibular grafts.

After 3 years, local relapse occurred, confirmed by radiological examination (**Fig. 1.b**.), by lab tests, elevated alkaline phosphatase serum levels, and by immunological tests, low anti-sarcoma antibodies serum levels.

**Table 1:** THE CASES WITH MALIGNANT BONE TUMORS

Nr	Name	Sex	Tumor type	Tumor location	Age at diagnosis	Age at last evaluation	Surgical treatment	Indication	External fixator type	Complications	Results	Survival
1.	U.L.	M	Juxtacortical osteosarcoma	Left femoral diaphysis	20 years	34 years	1990 – tumor biopsy 1995 – en bloc resection + autogenous fibular graft reconstruction 1998 – relapse; graft excision, modular diaphysis prosthesis made of hidroxiapatita on gamma nail splint 1999 – osteitis due to bioelectrolisis with fistula and partial deterioration of the mounting. Ablation of the mounting and external fixator implanting “on wait” 1999 - (after 3 weeks) – massive autogenous tibia graft on external fixator 2000 – application of controlled electromagnetic field for 6 months	To sustain the osteitis treatment	Ortofix adjustable	10 cm shortening	Very good statics and locomotion 14 years after surgery. Socially integrated.	18 years
2.	C.T	F	Osteo-genic osteosarcoma	Distal metaphysis of right femur	14 years	17 years	2001– epiphysiolysis, autogenous graft – boiling sterilization	Intraoperative epiphysiolysis	Ortofix adjustable	-	Limited knee mobility after 3 years. Socially integrated	7 years
3.	M.R	F	Ewing sarcoma	Distal third of right femur	14 years	16 years and 6 months	2002 – Intraoperative epiphysiolysis, autogenous graft sterilizated by boiling, plate and screws osteosinthesis 2003 – right lower limb disarticulation	Intraoperative epiphysiolysis	Ortofix adjustable	Local relapse, lung metastases	Difficult gait and crutches are needed after 6 months	Deceased after 18 months in 2003
4.	L.P	F	Giant cell tumor – the fourth stage	Distal third of left femur	19 years	22 years	1999 – tumor resection (13 cm bone defect) – proximal femur and distal tibia corticotomy, bone transport	Bone transport for remaining defect reconstruction	Ilizarov Complexe	Articular and epiphysis extent. Infection on a wire	The healing of arthrodesis	9 years
5.	G.G	F	Chondrosarcoma	Distal third of left femur	16 years	22 years	1998 – oncological en bloc resection, allograft, plate and screws osteosinthesis 1998 – allograft infection, allograft resection, gentamicin pearls in remaining infected cavity and external fixator implanted 1999 – knee modular prosthesis	To sustain the allograft infection treatment	Ortofix adjustable	Allograft infection	Normal statics and locomotion after one year, socially integrated. Local relapse and lung metastases after six years	Deceased after 6 years in 2004
6.	S.T	F	Osteolytic osteosarcoma	right tibial proximal metaphysis	16 years	18 years	2001 – epiphysiolysis, tumor resection , allograft, plate and screws osteosinthesis 2001 – distal pin ablation and proximal replacement with other pin	Slow and progressive epiphysiolysis	Monoplanar Triaxial Mixed	Infection on diaphysis pin	Integrated allograft, normal statics and locomotion. Socially integrated	7 years
7.	N.G	M	Ewing sarcoma	left humeral diaphysis distal half	14 years	21 years	1997 – oncological en bloc resection, autogenous fibular graft 1998 – local bone compacting	-compacting pseudarthrosis on graft -to sustain autogenous graft	Ortofix adjustable	Graft fracture after 8 months. Limited arm abduction after one year	Autogenous graft integrated, normal shoulder and elbow mobility after 7 years	10 years
8.	C.N	M	Ewing sarcoma	left tibial diaphysis	15 years	15 years and 6 months	2003 – external fixator implanted	comfort	Ilizarov adjustable	Fracture	Comfort (no cast required)	Deceased after 6 months in 2004

On surgery, we practiced tumor relaps resection, grafts resection and a modular diaphyseal prosthesis made out of a hidroxiapatite implant on a gamma nail splint (**Fig. 1.c**.).

One year after diaphyseal prosthesis implantation, due to bioelectrolysis, osteitis occured (probably due to idiosyncrasy, followed by fistulation). Immunohistochemistry study showed elevated Fe, Mg and C levels in the bioelectrolysis liquid.

On reintervention we practiced prosthesis and gamma nail ablation. We also practiced sample prelevation from the ends of the remaining fragments in order to perform pathological anatomy and immunohistochemitry exams. We used an external fixator, implanted “in abeyance”, in order to decide whether a femoral endoprosthetic reconstruction or a bone reconstruction was needed. The pathological anatomy exam did not show neoplastic elements, so we decided to practice on external fixator a massive autogenous tibial graft, representing 1/3 of homolateral tibial volume.

The use of bone grafts (fibular, tibial, costal, etc.) longer than 5 centimeters leads to pseudoarthrosis or late consolidation (**Fig. 1.d**.), which may favour fractures. 

In order to sustain these grafts, external fixator implant is very useful in avoiding their fracture in bone resorption areas. The delayed consolidation of the joint between the graft and the distal femoral end was treated by compaction on the external fixator.

Because of the many interventions due to local relapses, a relatively high (10 centimeters) shortening occurred, but it has been well tolerated: there are 14 years after the first surgery and the patient could walk after only 4 months postoperative. We applied as a treatment controlled electromagnetic field for 6 months. After 14 days, since the intervention, we alternatively applied a 0.02 T electromagnetic field, half an hour proximal and half an hour distal, twice a day (in the morning and in the evening). 

Postoperative, at two months and four months, respectively, the control radiographs, after applying of the electromagnetic field, showed signs of bone reorganization in the proximal fragment and no relapse.

In 2004, at the time of the last evaluation, 14 years after the first surgery, we ascertained very good statics and locomotion and complete social integration of the patient. (**Fig. 1.e**.).

**Case 2**

Patient C.T., female, aged 14, transferred from another hospital, is admitted with the diagnosis of osteogenic osteosarcoma of the distal metaphysis of right femur, which has been established by pathological anatomy exam. The diagnosis is confirmed on reexamining the samples. On surgery we implanted an external Ortofix fixator using slow and progressive epiphysiolysis. After 3 weeks, on surgery we practiced oncological “en bloc” tumor resection and an allograft implant fastened by 2 Ender rods, plate and screws. At the age of 17, after 3 years of survival, the patient was integrated in school and had limited knee mobility.

**Case 3**

Patient M.R., female, aged 14, is admitted with the diagnosis of tumor of the distal third of right femur. We practiced surgery in order to prelevate biopsy. The pathological anatomy and immunohistochemistry exams established the diagnosis of Ewing sarcoma.

We established the therapeutical strategy, with chemotherapy, radiotherapy and, after 6 weeks we intended to perform surgery. The hesitation of the family members and their attempts to find a doctor to heal the girl, determined a very late intervention, the surgery being performed one year after the biopsy. The MRI exam performed before surgery showed tumor extension to the soft tissues and diaphysis, without invasion of the physis. Surgery consisted of intraoperative epiphysiolysis on Ortofix external fixator, followed by “en bloc” oncological resection and tumor sterilization by thermal procedure. 

Subsequently, we practiced plate and screws osteosynthesis.

She could walk with the aid of crutches one month later. One year after surgery, local relapse and lung metastases occurred. On surgery, we practiced right lower limb disarticulation. The patient died three months after disarticulation, one year and six months after tumor resection and two years and six months after biopsy. 

**Case 4**

Patient L.P., female, aged 19, complained of pain in the left knee 8 month before, next she noticed the swelling of the thigh, in the distal third of the femur, and the valgus deviation of the knee. The knee radiograph showed an expansive tumor in the distal femoral metaphysis and diaphysis, with invasion of the knee joint and tibial diaphysis. The aspect was typical for giant cell tumor, with extended area of osteolysis, flourishing aspect (“soap bubble”), bone washing-out and the presence of disorganized trabeculae that determined tumor septation.

The early arterial phase of the angiography showed neovascularity in the diaphysis and also in the surrounding soft tissue. The late venous phase shows tumor extension and its limits.

During surgery, the extemporaneous histological examination confirmed the diagnosis of an aggressive giant cell tumor, of fourth stage. We practiced tumor resection, with the excision of eight centimeters of the femoral distal end, of the knee and of five centimeters of the tibial proximal end. We implanted a complex adjustable Ilizarov external fixator. We also made a double corticotomy, femoral and tibial, respectively.

After tumor resection, a total bone deficit of thirteen centimeters resulted, which could have been replaced by modular knee prosthesis, costing about 12.000 euro. For financial reasons, we used bone transport, with the disadvantage of a complex fixator implant for 202 days. The healing index was 15.6. 

The long fixator maintaining time led to infection around two of the wires, which have been removed and replaced by transosseous osteosynthesis at different points. For three months, we used a fixed knee orthesis. After one year, the patient could walk again, with the knee in fixed extension. On her revisit, three years after surgery, we ascertained the consolidation of the arthrodesis and the distraction points.

**Case 5**

Patient G.G, female, aged 16, has been admitted with the diagnosis of a tumor in the distal third of the left femur. The diagnosis of chondrosarcoma has been established by anamnesis, clinical examination, imaging data, angiography and immunohistochemistry. The surgery consisted in oncological “en bloc” tumoral resection, allograft implant and plate and screws osteosynthesis. After 45 days, allograft infection occurred and we proceeded to an Ortofix external fixator implant, allograft resection and the remaining cavity was filled with gentamicin pearls.

After draining the infectious process, in 1999, on request of the patient and family members, we implanted modular knee prosthesis, because there was no contraindication of this treatment. The patient could walk ten days after surgery. One year postoperative the statics and locomotion were normal and the patient was rapidly integrated to school.

Six years after the first surgery, the patient required a new evaluation because she noticed the swelling of the knee. We ascertained local relapse and lung metastases. She refused amputation and lung metastases excision. The patient died at the age of 22, after six years of survival.

**Case 6**

Patient S.T., female, aged 16, has been admitted with the diagnosis of a tumor in the proximal metaphysis of the right tibia. After performing the radiological protocol of investigation, we established the diagnosis of osteolytic osteosarcoma, without lung metastases. The MRI examination did not show invasion of the physis or the epiphysis.

We proceeded to slow and progressive epiphysiolysis on Monoplanar Triaxial external fixator. After epiphyseal lift up, we proceeded to tumor resection on an external fixator, allograft, plate and screws osteosynthesis. While maintaining the external fixator, we ascertained infection on a diaphyseal pin. We extracted the infected pin and replaced it downwards with other pin. 

On the last evaluation, at the age of 18 (two years after surgery), the patient was integrated to school. The radiograph showed the complete integration of the allograft. Nowadays the clinical evolution is favourable.

**Case 7**

**Fig.2:**
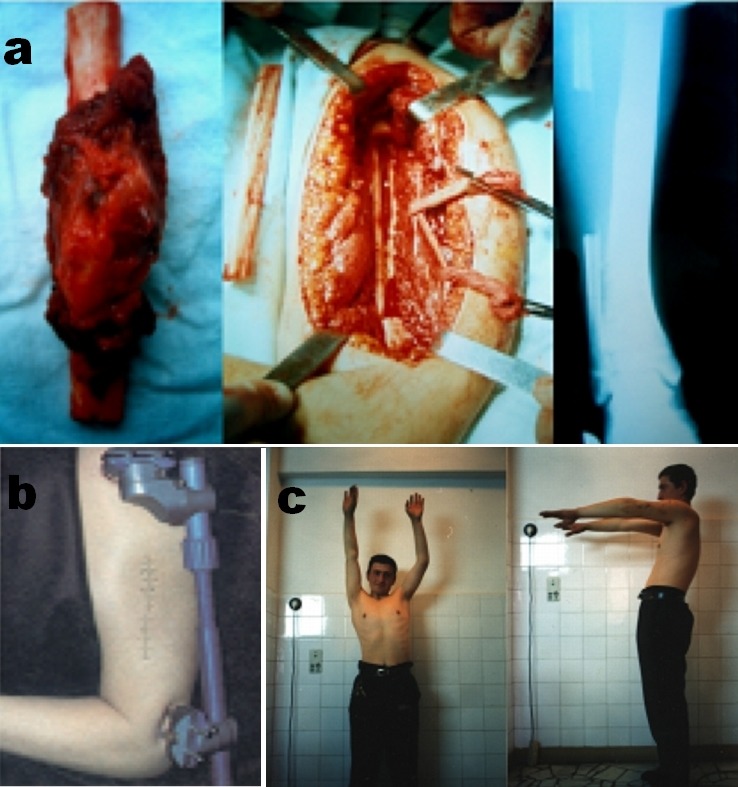
a. Intraoperative aspects after oncological resection and fibular bone graft harvest used in osteoplastic reconstruction of the humeral diaphysis. b. Image of the operated arm and of the fixator meant to ensure the fibular graft. c. The integrated fibular bone graft allowed active movement, limited to a 45 degrees abduction, one year later.

Patient N.G., male, aged 14, has been admitted with the diagnosis of a tumor in the distal half of the left humeral diaphysis. After performing standardized investigations, we established the diagnosis of Ewing sarcoma. After six weeks of chemotherapy and ten cobaltotherapy sessions (400 rad/session), we performed surgery with oncological “en bloc” tumor resection and bone reconstruction with autogenous fibular graft (**Fig. 2.a**.) on an Ortofix external fixator (**Fig. 2.b**.).

On periodic check performed after one, two and three years respectively, we noticed the progressive graft integration (**Fig. 2.c**.).

Eight months postoperative we ascertained a fracture of the medium third of the graft, which has been treated and cured by local bone compacting using the fixator. The last evaluation was performed after seven years of survival and did not show local relapse or metastases. 

**Case 8**

Patient C.N., male, aged 15, is transferred from an oncology clinic, where chemotherapy and radiotherapy were performed for a non-displacement fracture in the medium third of the calf, in an area of rarefied bone. After evaluation we proceeded to an Ilizarov external fixator implant to avoid further fracture displacement, in order to continue radiotherapy. This treatment did not require cast immobilization and the patient could walk, which was very important for his comfort. We intend to perform surgery after preoperative oncology treatment.

## Results

The eight patients, five females and three males, were aged between 14 and 20 years and the mean age was 16. Three of the eight patients died. One of them, with an advanced form of Ewing sarcoma and a pathological fracture, died after six months. The other, also with Ewing sarcoma, died after one and a half year postoperative because of the unjustifiable temporization of the surgery. The third, with chondrosarcoma and allograft infection treated by allograft resection and filling the infected cavity with gentamicin pearls and with a modular knee prosthesis implanted after that, died six years postoperative. Five of the patients survived in good conditions, with good statics and locomotion, socially integrated. The sample has no statistical significance because of the small number of cases. The external fixator implanting decision was made according to clinical evolution and complications occurrence. However, we can mention that the survival rate was 62.5% (5 patients of 8). In each case the survival was above 7 years. The survival below five years was registered in 25% of all cases (2 patients), the survival between 5 and 10 years - in 12.5% of all cases (1 patient) and the survival above 10 years - in 62.5% of all cases (5 patients). One of the patients who survived more than 5 years, died 6 years postoperative, because of the neoplastic impregnation syndrome, with bone tumor relapse and lung metastases.

Generally, bone tumors ***complications*** are very serious, imposing laborious surgical interventions or a conservative attitude and supportive treatment to maintain vital functions by the time of death. In our patients we registered the following postoperative complications: pathological fracture (one case), bone infection (three cases), limb shortening (one case), tumor extension (one case), relapse (one case), and metastases (two cases):

- Shortening of the affected limb – 10 centimeters. This complication occurred because of the many surgical interventions, necessary in a case of juxtacortical osteosarcoma with local relapse, osteitis, due to bioelectrolisis, followed by an autogenous tibial graft implant.

- Tumor extension from the distal femoral metaphysis to the epiphysis and transarticular to the tibia is a rare complication, which occurred in a case of fourth stage giant cell tumor.

- Local relapse – occurred in two cases. The first case was a chondrosarcoma and the tumor relapse was accompanied by another complication – lung metastases. Shortly after relapse, the patient died, six years postoperative. The second case, a patient with juxtacortical osteosarcoma, local relapse occurred on the two autogenous fibular grafts of 30 centimeters. After relapse we excised the grafts and implanted a modular diaphyseal prosthesis made of hidroxiapatite on a gamma nail splint.

- Infection – has been noticed in three cases. In two of these cases it was a minor complication, infection of the transosseous fixing wires. In the first case we removed the infected wire and replaced it with another wire to maintain the stability of the mounting. In the second case we simply removed the infected wire. In both cases, the infection was healed. In the third case, it was a major infection of the allograft. We proceeded to allograft resection and the remaining cavity was filled with gentamicin pearls. After one year, the infection was healed and we implanted a modular knee prosthesis. The patient died five years after modular prosthesis implant, because of local relapse and lung metastases.

- Pathological fracture– occurred in a case of Ewing sarcoma presenting in an advanced stage. For patient comfort and in order to perform cobaltotherapy we proceeded to an external fixator implant.

- Lung metastases – occurred in two cases.

## Discussion

Malignant bone tumors have a great extent tendency. First, invasion tends to destroy the metaphyseal marrow and to extend to the medullary cavity. As a rule, the diaphyseal medullar cavity invasion corresponds to the appearance of metaphyseal osteolysis areas and to the distruction of the cortical bone limiting the eccentric tumor.

Physeal invasion occurs after one or both cortical bones of the metaphysis are destroyed. The physis represents a relative obstacle against tumor extension. Tumor extension beyond physis represents epiphyseal invasion. Defining the epiphyseal invasion is very important for oncologic surgery. If the tumor does not cross beyond thephysis, it is possible to save the epiphysis and perform bone reconstruction (**Table 2**). If the tumor extends beyond it, it is compulsory to perform oncological “en bloc” tumoral resection and modular prosthesis implant (**Table 2**). Tumor extension to the soft tissues must be evaluated for each case, so the oncological resection should preserve certain parts of the extensor and flexor muscle, making possible the mobilization of the joint.

Tumor extension involveing the vascular and nervous bunch, as a habit require amputation and rarely vascular or nervous graft is indicated.

A significant tumor extension to the soft tissues, with neoplastic impregnation of the skin requires amputation, too.

The use of external fixators, according to the extension degree, is limited. The correlation between extension and the type of the external fixator used only depends of the bone tumor extension.

Today there is a special interest in the use of external fixators in tumor resection and bone reconstruction technique.

**Table 2:** Tumor types for which we implanted external fixators and we practiced reconstructions and prosthesis implanting

Histological type	Prostheses	Reconstructions	Total
Osteosarcoma	1	2	3
Ewing sarcoma	0	3	3
Chondrosarcoma	1	0	1
Giant cell tumor fourth stage	0	1	1
Total	2	6	8

***Periodical evaluation***

*Malignant tumors evaluation* should be much more complete. The first *preoperative diagnostic evaluation* is performed before open biopsy. It consists of laboratory testing: red blood cells sedimentation rate, lactic dehydrogenase, creatin kinase, alkaline phosphatase, anti-vimentin, anti P100 antibodies (**Table 3**) and anti-sarcoma antibodies serum levels. Imagistic evaluation consists, beyond customary radiographs, computed tomography, magnetic resonance imaging, bone scintigraphy and angiography.

**Table 3:** Serum antibodies

	Anti-vimentin	Anti P100	Other
Osteosarcoma	+	-	-
Ewing sarcoma	+	+/-	-
Chondrosarcoma	+	-	-
Aggressive giant cell tumor fourth stage	+	-	-

Usually, these investigations are performed before open biopsy. The therapeutic strategy consists of 4-6 weeks of preoperative chemotherapy for the majority of bone tumors. The treatment scheme varies with the tumor histological type. 

For each patient with malignant bone tumor the preoperative diagnostic evaluation consisted of every laboratory and imagistic examinations mentioned above.

*The preoperative evaluation after chemotherapy* is selective, being reserved only for tumors with great extension, that could excessively invade the soft tissues and also for metaphysis tumors, extended to the growing cartilage limit. For the first case it is important to establish whether the remaining muscle after oncological resection would ensure joint mobility. For the second case it is important to decide whether the epiphysis can be saved, so bone reconstruction is possible, or the epiphysis must be excised, so modular prosthesis is needed. In order to establish the therapeutic strategy, a computer tomography scan or a magnetic resonance imaging study must be performed.

Thoracal pleuralpulmonary radiograph is a usual preoperative investigation, meant to ascertain whether lung metastases are present or not. If there are radiopaque processes in the pulmonary hila, that are difficult to diagnose, it is necessary to perform a pulmonary computer tomography scan. If there are lung metastases, the therapeutic strategy needs to be changed.

The angiography performed during the diagnostic evaluation, or, if necessary, after chemotherapy, is very important in ascertaining tumoral extension to the soft tissues and in establishing the relationship between the tumor and adjacent neovascular structures. The early arterial phase shows active neovascularity around the tumor, extended to the soft tissues. The late venous phase shows the intrinsic blood vessels of the tumor, createing the aspect of “red tumor”. The “red tumor” image delineates the area of the sarcomatous tumor that must be radically excised, in order to save the affected limb. 

*Postoperative evaluation* of the eight patients in our study consisted in radiologic and laboratory tests. Radiographs were performed in order to ascertain graft integration, the stability of the prosthesis and possibly the occurrence of local relapse or metastases. Laboratory tests were performed in order to monitorize tumor evolution. We determined: red blood cells sedimentation rate, alkaline phosphatase, creatin kinase, lactic dehydrogenase and anti-sarcoma antibodies serum levels. The high alkaline phosphatase serum levels show osteogenesis of the malignant tissue. The high levels of this enzyme correlate with the degree of neoplastic osteoblasts activity and with the size of the tumor, too. Dynamic enzyme evaluation is useful for osteosarcoma evolution monitoring. The serum level of this enzyme decreases almost to normal after tumor excision and increases in case of metastases or relapse.

Anti-sarcoma antibodies serum levels increase after tumor resection and decrease when relapse or metastases occur.

The patients with malignant bone tumors have been continuously monitorized, every 3-4 months during the first year and twice a year in the following period. Lung metastases occurrence imposes patient reevaluation in order to establish the indication of bone or lung metastases excision. If osseous metastases considerably weakened the bone or pathological fractures occurred, bone reconstruction for patients comfort is performed. 

## Mounting types

The orthopaedic treatment of malignant bone tumors implies complex mountings, difficult to be configured before surgery is performed. These mountings are adapted to each particular case and usually bone transport is needed, in order to compensate the remaining osseous defect after oncological en bloc resection. Only one of our patients needed a complex mounting. This mounting has been used for a fourth stage giant cell tumor with transarticular extension. It involved two fragments transport (femoral and tibial respectively). Complex mountings are usually made on Ilizarov or Monticelli external fixator.

Of all 16 mounting types, the most frequent were adjustable (used in 8 cases), less frequent used mounting types were mixed (4 cases), rigid (3 cases) and complex mounting was used only for one case.

These mountings were made on different types of external fixators: Ortofix – 6, Monoplanar Triaxial – 3, Ilizarov – 2, Fixano – 2, Shearer – 2 and Monticelli – 2.

**Table 4:** Mounting types- technical considerations

Mounting type	Benign	Malign	Total
Adjustable	2	6	8
Rigid	3	-	3
Mixed	3	1	4
Complex	-	1	1
Total	8	8	16
			

Monoplanar external fixator, all-round or circular, can be used in different situations:

- Preoperative – before oncological “en bloc” resection

This indication has been useful for a patient with Ewing sarcoma in an advanced stage, complicated with a pathological fracture. The external fixator conferred comfort because to the patient, he could walk and it permitted preoperative radiotherapy, too. Using this fixator we avoided patient immobilization in bed and we excluded the risk of metastases implied by other interventions.

- Intraoperative – for extemporaneous epiphysiolysis

Intraoperative epiphysiolysis is performed before bone reconstruction. We used this strategy for a Ewing sarcoma. In this case after epiphysiolysis we implanted an autogenous graft that had been sterilized by boiling and then we performed plate and screws osteosinthesis.

- Postoperative

We used external fixators for the following situations: 

a) External fixator to sustain autogenous graft

This indication has been very useful for the patient with juxtacortical osteosarcoma. In this case we implanted a 30 centimeters autogenous homolateral tibial graft, sustained by an Ortofix fixator. A 14 years old patient with Ewing sarcoma had the same indication. In this case, after humeral diaphysis oncological resection we implanted an embedded autogenous fibular graft.

b) External fixator as a separate technique for slow and progressive epiphysiolysis

After studying 24 bone samples from patients with metaphysis or diaphysis osteosarcoma, Canadell noticed that in half of these cases the tumor was not extended to the growing cartilage or to the epiphysis. In order to determine tumor extension to the growing cartilage, we used radiographs, computed tomography and angiography. MRI is the perfect method to determine the extension to the growing cartilage.

In one case, after growing cartilage was decollated using a distraction regimen of 1-2 milimeters every day, we excised the tumor and then we implanted an autogenous graft sterilized by boiling, which has been stabilized by the same external fixator. In another case, after epiphysiolysis we excised the tumor and then we implanted a graft stabilized by plate and screws. Osteoarticular allografts are useful for tumors with certain anatomical localization. The size of these grafts must correspond with the size of the excised bone. When using allografts, the recovery period is long. Severe complications may occur during the first three years after surgery. Mankin et al. report 14% of these complications as infections, 10% as fractures, 9% as pseudoarthroses and 3% as joint instability. The implant failure is estimated to 33% after 60 months follow up (Joffe et al.).

c) Bone transport used to compensate the remaining osseous defect

For a 19 years old patient with a fourth stage giant cell tumor extended transarticulary, we performed tumor resection leading to an osseous defect of 13 centimeters that had been reconstructed by double (tibial and femoral) bone transport. 

d) External fixator used for local bone compacting in case of graft fracture or pseudoarthrosis

In the patient with embedded autogenous fibular graft, graft fracture occurred after 8 months. The adjustable mounting on Ortofix fixator allowed bone compacting at the fracture site, leeding to the healing of this complication.

e) External fixator used to sustain allograft infection treatment and prosthesis

Limb reconstruction refers to every technique used to rehabilitate bone continuity and also joint mobility. In case of allograft infection, such as in our patient with osteosarcoma, we excised the allograft and filled the remaining cavity with gentamicin pearls in order to preserve the length of the limb and maintain it by external fixator implant. After the infection was healed, a modular knee prosthesis was implanted.

## Conclusions

After studying the 8 malignant bone tumors for which external fixators were used, we came to the following conclusions. For malignant bone tumors, external fixators are used in the following circumstances:

a) Preoperative – in case of secondary fracture, for patient comfort and in order to perform radiotherapy, before oncological en bloc resection;

b) Intraoperative – for extemporaneous epiphysiolysis, followed by tumor resection and bone reconstruction;

c) Postoperative

• to sustain autogenous graft; external fixator supports bone reconstruction. Charging the affected lower limb (static and dynamic loading) facilitates osteogenesis and grafts integration, allows compacting in case of late consolidation between graft and bone extremities and also avoids fractures in high risk bone resorption areas (with extensive osteoporosis);

• to perform slow and progressive epiphysiolysis;

• for remaining defect reconstruction by bone transport;

• for local compacting in case of graft fracture or pseudoarthrosis;

• to sustain allograft infection treatment; in order to maintain limb length and to perform prosthesis implant after infection healing.

Mixed or complex adjustable mountings are used for malignant bone tumors.
